# Review immune response of targeting CD39 in cancer

**DOI:** 10.1186/s40364-023-00500-w

**Published:** 2023-06-07

**Authors:** Yao Liu, Zhongliang Li, Xiaoguang Zhao, Jing Xiao, Jiacheng Bi, Xian-Yang Li, Guokai Chen, Ligong Lu

**Affiliations:** 1 Guangdong Provincial Key Laboratory of Tumor Interventional Diagnosis and Treatment, Zhuhai People’s Hospital, Zhuhai Hospital Affiliated with Jinan University, Zhuhai, 519000 Guangdong P.R. China; 2 Institute of Translational Medicine, Faculty of Health Sciences, University of Macau, Taipa, Macau China; 3 CAS Key Laboratory of Quantitative Engineering Biology, Shenzhen Institute of Synthetic Biology, Shenzhen Institute of Advanced Technology, Chinese Academy of Sciences, Shenzhen, China; 4 Department of R&D, OriCell Therapeutics Co. Ltd, No.1227, Zhangheng Rd, Pudong, Shanghai, China

**Keywords:** CD39, Adenosine pathway, Immune checkpoint blockade (ICB), PD-1

## Abstract

The ATP-adenosine pathway has emerged as a promising target for cancer therapy, but challenges remain in achieving effective tumor control. Early research focused on blocking the adenosine generating enzyme CD73 and the adenosine receptors A2AR or A2BR in cancer. However, recent studies have shown that targeting CD39, the rate-limiting ecto-enzyme of the ATP-adenosine pathway, can provide more profound anti-tumor efficacy by reducing immune-suppressive adenosine accumulation and increasing pro-inflammatory ATP levels. In addition, combining CD39 blocking antibody with PD-1 immune checkpoint therapy may have synergistic anti-tumor effects and improve patient survival. This review will discuss the immune components that respond to CD39 targeting in the tumor microenvironment. Targeting CD39 in cancer has been shown to not only decrease adenosine levels in the tumor microenvironment (TME), but also increase ATP levels. Additionally, targeting CD39 can limit the function of Treg cells, which are known to express high levels of CD39. With phase I clinical trials of CD39 targeting currently underway, further understanding and rational design of this approach for cancer therapy are expected.

## Introduction of adenosine pathway in cancer

**Fig. 1:**
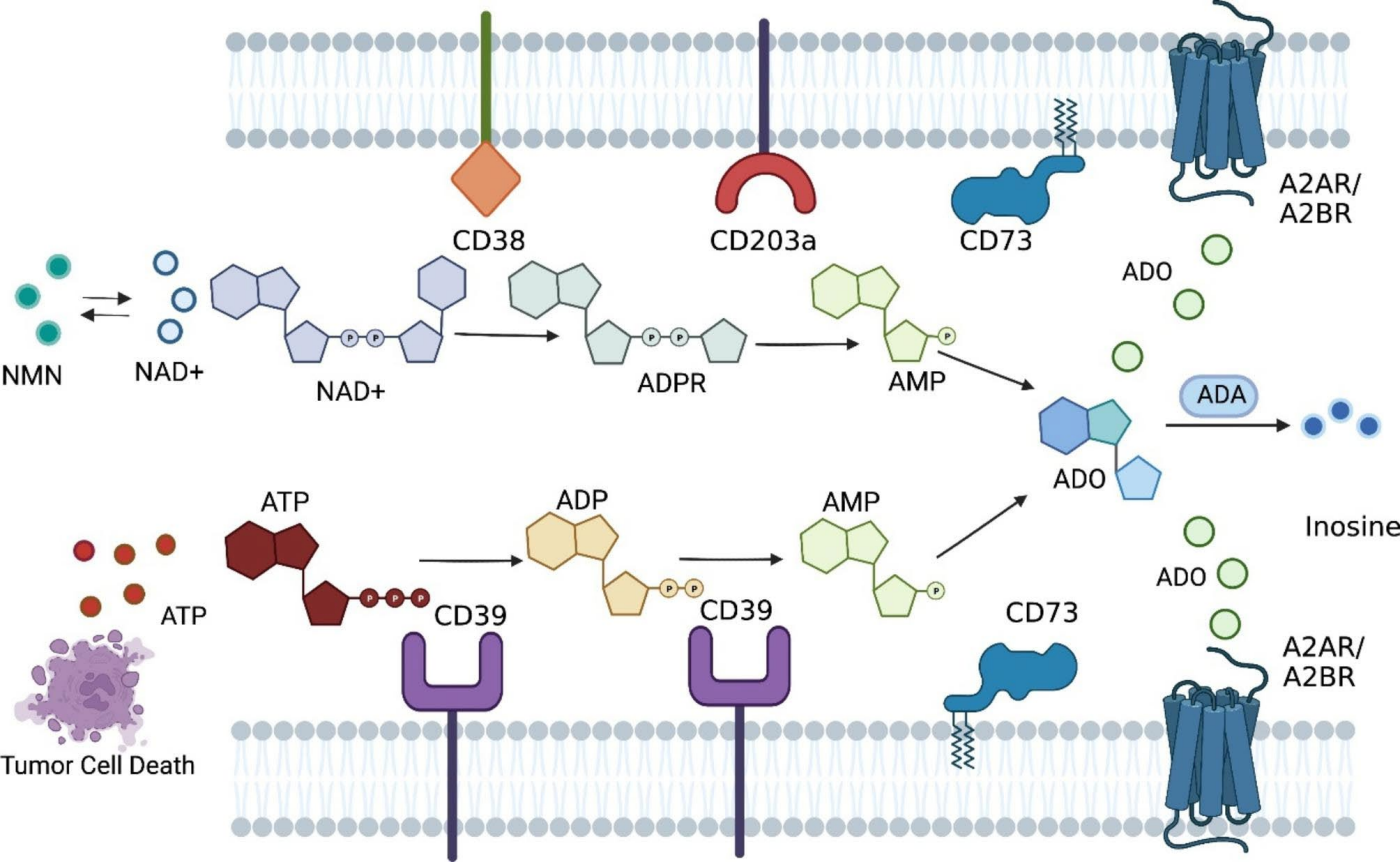
ATP-ADO pathway. The ATP-ADO pathway is critical in regulating immune responses in the tumor microenvironment. Two primary pathways are involved in generating adenosine (ADO): the CD39/CD73 pathway and the CD38/CD203a/CD73 pathway. In the CD39/CD73 pathway, CD39 degrades ATP into ADP and AMP, which is further degraded into ADO by CD73. In the alternative CD38/CD203a/CD73 pathway, nicotinamide adenine dinucleotide (NAD+) is sequentially degraded into ADPR, AMP, and ADO. Adenosine binds to adenosine receptors (i.e., A2AR and A2BR) and inhibits immune cell activation. Additionally, adenosine aminohydrolase (ADA) can convert ADO into inosine. Furthermore, the CD39/CD73 pathway is the predominant mechanism for ADO generation in the TME, and targeting CD39 has emerged as a promising strategy for cancer therapy

Adenosine is a key effector molecule that regulates both innate and adaptive immunity. Adenosine functions through type 1 purinergic receptors (A1, A2A, A2B, and A3), which belong to G protein coupled receptors (GPCR) [1]. A1 and A3 receptors inhibit adenylate cyclase and cAMP production, and thus promote immune cell activity. In contrast, A2A and A2B receptors, which are abundantly expressed in various immune cells, such as myeloid cells and lymphocytes, are associated with immunosuppression by triggering intracellular cAMP accumulation. A1 and A2A receptors have high affinity to adenosine and play a dominant role in physiological conditions, while A2B and A3 receptors have low adenosine affinity and are critical in some pathological conditions such as malignancies [2–6]. Adenosine can be generated by adenosine triphosphate (ATP) through the CD39/CD73 pathway, which is particularly prevalent in the tumor microenvironment (TME) [6–9]. The major energy currency ATP, under normal or physiological conditions, is mainly located inside the cell, with a concentration of about 1 ~ 10 mM, while the extracellular ATP (eATP) concentration is at a very low level (10 ~ 100 nM). However, in the TME, eATP can be released by stressed or dying cells and provides inflammatory signals that are critical for effective innate and adaptive immune responses [10–12]. The hypoxia and inflammatory environment in tumors also can induce the expression of CD39 and CD73, two key ecto-enzymes in the ATP-adenosine pathway [13–15]. CD39 converts eATP to adenosine diphosphate (ADP) and adenosine monophosphate (AMP), whereas CD73 hydrolyzes AMP into adenosine. Hence, adenosine can be upregulated from low levels (around 1 µM) under normal conditions to high levels (> 100 µM) in the TME, resulting in an immune-suppressive environment and promoting tumor development [10, 16]. Alternative pathways such as CD38/CD203a/CD73 and ALP also can produce adenosine in the TME from NAD^+^ and ATP, respectively. There are currently studies suggesting that this non-canonical adenosine-generating pathway may also play an important role in cancer [17–19]. It is worth mentioning that the ENPP1-CD73 pathway as an alternative adenosinergic loop that could allow cancer cells to evade CD39-targeted therapeutics. However, the CD39/CD73 pathway is considered to be the major source of adenosine in the TME (Fig. 1) [1, 5, 6, 10].

CD39, as well as the downstream molecules of the ATP-adenosine pathway, such as CD73 and A2AR/A2BR, play critical roles in tumor growth. Their expression is usually elevated in the tumor components, including tumor cells and immune cells, and is associated with poor prognosis in several malignancies. Consequently, several clinical trials are being conducted to target the adenosine pathway for cancer treatment [7, 10, 14, 15]. CD73 and adenosine receptors were the primary targets focused by researchers [8, 20–22]. Recent clinical advances in CD73 blocking antibodies or antagonists and inhibitors of A2AR and A2BR have demonstrated the therapeutic potentials of modulating the adenosine pathway in cancer [8, 20, 23–25]. Targeting CD39 is also promising in cancer, with a general anti-tumor mechanism that rely not only on preventing the immune-suppressive adenosine level but also on the stabilization and accumulation of pro-inflammatory eATP to restore anti-tumor immunity. Blocking antibodies targeting CD39 are now being investigated for tumor control in clinical trials [10, 26–28]. Further understanding of the detailed mechanisms of action of CD39 antagonists, in particular how the immune components responding to CD39 blocking antibody, is now a priority for rational design of targeting CD39 in cancer [10].

### CD39 in tumor

CD39 is an ectonucleoside triphosphate diphosphohydrolases (encoded by ENTPD1 gene) and a member of the NTPDase family comprising of four cell-surface members (NTPDase1,2,3 and 8), and NTPDase4,5,6 and 7 being expressed as intracellular enzymes [29, 30]. With the ability to regulate purine metabolism, all the family members, especially CD39, CD73, ENPP1 (also known as CD203a), and CD38, are potential tumor immunotherapy targets [12, 31–33].

Human CD39 is a 510-amino-acid protein with 7 N-glycosylation sites and 11 cysteine residues. Structurally, CD39 includes two transmembrane domains, a small cytoplasmic domain, as well as a large extracellular region enriched with hydrophobic residues. The cytoplasmic domain has both N-terminal and C-terminal sections, whereas the extracellular domain contains five ATPase conserved areas [31].

Although CD39 is primarily characterized as an Ecto-(Ca^2+^, Mg^2+^)-apyrase [34], it is also a Treg cell marker and is widely expressed in a variety of tissues and organs, particularly in endothelial cells, fibroblasts and numerous subsets of immune cells, including B cells, Treg cells, macrophages, and effector T cells [10, 13, 31, 35]. CD39, CD73 and adenosine receptors are upregulated in pathological and physiological conditions such as tissue growth, tissue damage, tissue remodeling and hypoxia [26, 36–38]. In addition, CD39 is also upregulated in response to chronic inflammation (e.g., tumor necrosis factor-alpha (TNF-α), interleukin (IL)-6, IL-27, and T cell exhaustion) [10, 39, 40]. Moreover, oxidative stress and aryl hydrocarbon receptor (AHR) have been demonstrated to upregulate CD39, which governs the function of tumor-associated macrophages [41].

**Fig. 2:**
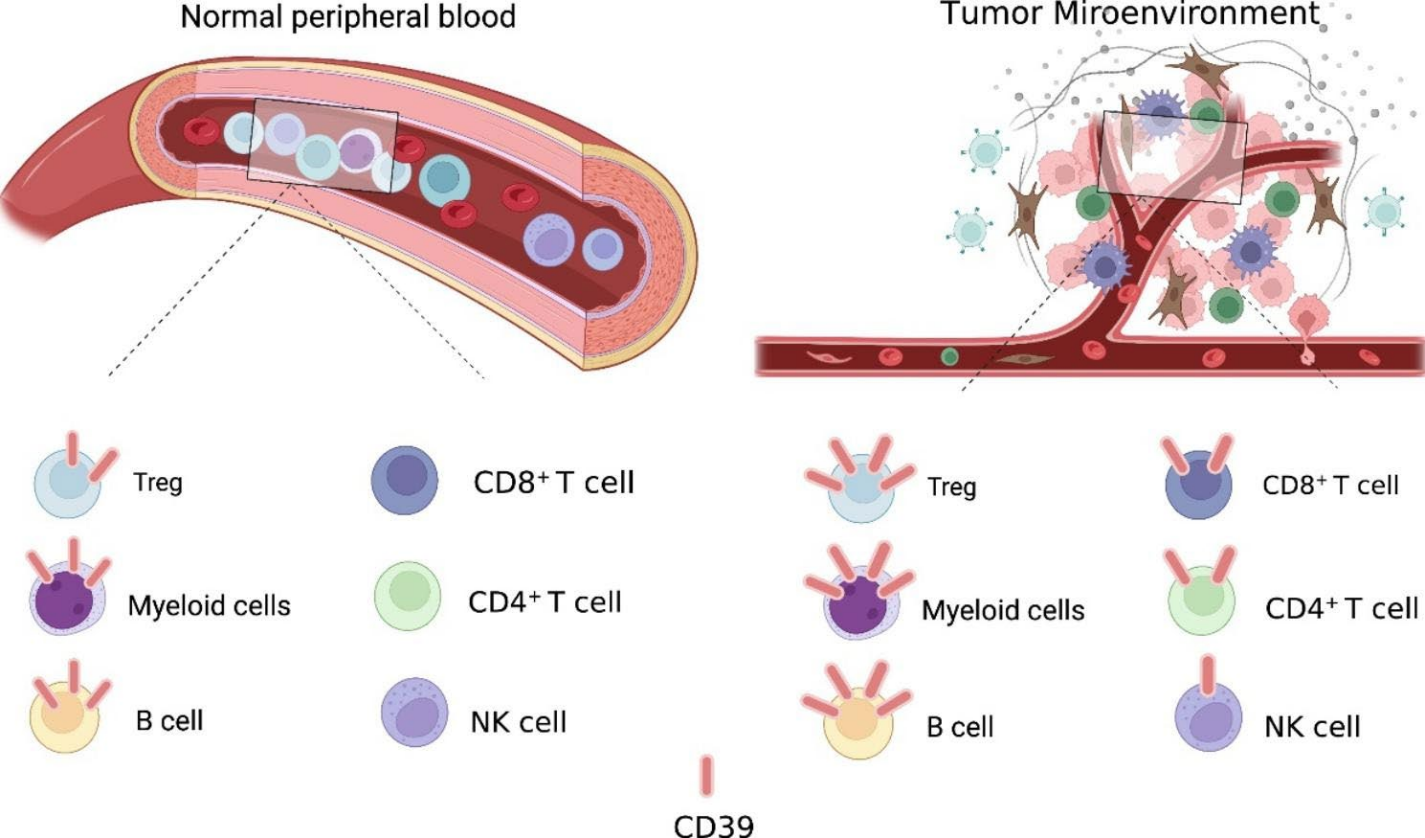
CD39 expression in immune cells under different conditions. In normal peripheral blood cells, CD39 is primarily expressed on T regulatory cells (Tregs), myeloid cells, and B cells. However, in the tumor microenvironment (TME), CD39 expression is upregulated in most immune cell populations, including myeloid cells, Tregs, T cells, and NK cells. This increase in CD39 expression leads to the generation of an immunosuppressive microenvironment with high levels of adenosine, which can inhibit immune cell function and promote tumor growth

CD39 expression is increased in various human tumors, including melanoma, colon cancer, ovarian cancer, pancreatic cancer, kidney cancer, lung cancer, thyroid cancer, testicular cancer, sarcoma, lymphoma, and chronic lymphocytic leukemia [42–46]. In the TME, CD39 expression is enriched in vascular endothelial cell, fibroblasts, myeloid cells, T regulatory cells (Tregs), tumor-specific T effector cells and NK cells (Fig. 2) [10, 12, 31]. In very rare circumstances, tumor cells can overexpress CD39 compared with neighboring normal tissues and cells [26, 47]. CD73 is also increased in human tumors, with frequent expression on tumor cells, endothelial cells, fibroblasts, and myeloid cells. Therefore, conversation from pro-inflammatory ATP to immune-suppressive adenosine is active in the TME due to increased expression of CD39 and CD73 in non-immune cells, immune cells, and tumor cells [14, 16, 48]. We will discuss the expression and function of CD39 in immune cells in the TME and how the immune cells respond to CD39 antagonists used to treat cancer in details in the sections below.

## CD39 and myeloid cells

### Macrophages

Macrophages co-express CD39 and P2 × 7, the eATP receptor [10, 26]. In the TME, macrophages secrete, hydrolyze, and respond to eATP via increasing expression of CD39 upon P2 × 7 activation [12, 49–51]. Consequently, blocking CD39 ATPase activity with antibody or inhibitor on macrophages dramatically boosts their production of IL-12 and TNF-α, while significantly lowering IL-10 secretion. In the LPS-induced sepsis mouse model, CD39-deficient mice consistently have increased levels of inflammatory cytokines. By contrast, suppressing macrophage -expressed P2 × 7 alleviates inflammatory cytokines production [52]. Therefore, CD39 could act as a “molecular switch”, that limits the balance of inflammatory and regulatory macrophage differentiation [10, 12, 31].

ATP released by dead or dying cells in the TME represents a “find me” signal, promoting the chemotaxis of macrophages, monocytes, and neutrophils. Despite the accumulation of extracellular ATP surrounding the cell surface enhances chemotactic signals by triggering P2Y2 receptors, sufficient exogenous ATP has been shown to block chemotaxis of macrophages, indicating that the chemotaxis process of macrophages is governed by eATP gradients [10, 31, 48, 53].

Increased eATP also stimulates macrophage phagocytosis via activation of P2 × 4 and P2 × 7 receptors [54]. In addition, increased levels of eATP lead to increased release of microvesicle from macrophages and monocytes, a major secretory pathway for rapid production of IL-1β and IL-18 which is mediated by P2 × 7 activation [26]. Moreover, microvesicles released from macrophages activate unprimed macrophages partially dependent on TLR4, inducing expression of costimulatory molecules CD80, CD86 and MHC class II, which in turn increases secretion of TNF-α from other macrophages [10, 12, 54].

Extracellular ATP is unstable in the TME and can be hydrolyzed immediately to adenosine by upregulated CD39 and CD73 in macrophages and other cells. It is well documented that adenosine directly suppresses macrophages by signaling through adenosine receptors, which suppresses macrophage differentiation and maturation, reduces expression of pro-inflammatory cytokines, and increases expression of immune-suppressive molecules, such as VEGF, arginase 1 and IL-10 [4–6, 55]. Hence, macrophages are potential positive responders for CD39 targeting by reversing their immune suppressive functions to immune activation components in cancer [26].

### Myeloid-derived suppressor cells

Myeloid-derived suppressor cells (MDSC) are accumulated in the TME and have been identified as a key player in the immune evasion mechanism of cancer cells [10]. CD39- and CD73-expressing MDSCs, which are induced by TGFβ and HIF1α, are often associated with tumor stage, node involvement and metastatic status within non-small-cell lung cancer (NSCLC) and colon cancer [56, 57]. MDSCs and CD39 expression on CD8^+^ T cells, in particular, predict the efficacy of immune checkpoint blockade (ICB) in patients with advanced NSCLC [57]. Consistently, tumor -associated MDSCs with high levels expression of CD73, CD39 and PD-L1 are characteristics of increased immunosuppressive activity ex vivo than myeloid cells circulating in peripheral blood [10, 12].

### Neutrophils

CD39 is also abundant in neutrophils. In mice, injection of ATP promotes neutrophil recruitment through a caspase-1/11-dependent mechanism. Consequently, CD39 hydrolyzes ATP and activate adenosine receptors to promote neutrophil chemotaxis [58]. However, controversial findings have demonstrated that LPS injection led to increased neutrophil trafficking to the lungs absence of CD39 [59]. In addition, non-hydrolysable ATP inhibits neutrophil chemotaxis, whereas exogenous hydrolysable ATP stimulates neutrophil chemotaxis [5, 10, 60]. Overall, these findings suggest that blocking CD39 may prevent neutrophil chemotaxis when neutrophils migrate to ATP-rich areas.

## CD39 and T cells

### CD4^+^ T cells

FOXP3^+^ Treg cells, an immune-suppressive CD4^+^ T cell subset with high CD39 expression, modulate immune activities through a variety of mechanisms, including the CD39-dependent production of adenosine [61–64]. CD39 represents a suppressive function marker in Treg cells. According to the data from CD39-deficient mice, Treg cells rely on CD39 for the suppressive activities in vitro and in vivo [31, 64, 65]. Consistently, CD39 expression on Treg cells restricts experimental colitis model induced by T cell transplantation [66]. Furthermore, in tumor metastasis models, CD39^+^ Treg cells in mice potently inhibit NK cell -mediated anti-tumor immunity [64, 67]. Human colon cancer -derived CD39^+^ Treg cells also suppress IFN-γ production and the in vitro proliferation of conventional T cells [67, 68].

Adenosine-stimulated signals may raise intracellular cAMP levels, leading to trans activation of ENTPD1 promoter, and thereby boosting and stabilizing the expression of CD39 in Treg cells. A decrease in the number of CD39^+^ Foxp3^+^ Tregs was observed in patients with multiple sclerosis (MS), indicating the importance of CD39 expression in Tregs in the management of inflammatory autoimmune diseases [69]. In contrast, circulating CD39^+^ CD25^+^ Treg levels increased in cancer patients, while low levels of CD39^+^ Tregs were associated with improved recurrence-free survival in melanoma patients [70]. In addition, the elevated levels of CD39 in tumor infiltrating Treg cells are usually accompanied by elevated levels of other inhibition/activation markers (i.e., PD-1, CTLA-4 and OX40) [49]. All these observations suggest that CD39, by stabilizing FOXP3^+^ Tregs, plays a key role in Treg-mediated tumor immunosuppressive response.

Type 1 regulatory T (TR1) cells, a second CD4^+^ subtype of regulatory lymphocytes, are induced from CD4^+^ T cells upon exposure to IL-27 [71]. TR1 cells, characterized by IL-10 production and the expression of LAG3 and CD49b, also utilize CD39 for immunosuppression [72, 73]. Interestingly, TR1 cells induced with IL-27 do not express CD73, implying that TR1 cells require the synthesis of adenosine in conjunction with CD73 expressed by other immune components such as effector T cells and DCs [53, 72]. Importantly, TR1 cells, which are FOXP3^–^, can be induced in the TME with a CD39-dependent manner. It is reported that eATP inhibits TR1 cell differentiation, whereas CD39 mediates TR1 suppressive activity via adenosine, indicating that TR1 could be a potential target for CD39 blocking therapy in cancer [72].

Th17 cells are significant drivers of chronic inflammation because they regulate autoimmunity and commensal microorganisms in the gut. Interestingly, CD39 expression confers suppressive function of Th17 cells via ATPase activity and related increased IL-10 production. In cancer patients, Th17 cells expressing CD39 can be used to predict poor clinical outcome. Th17 cells with low levels of CD39 and CD73, which generated in the absence of TGFβ, are endowed with anti-tumor functions [10, 12, 74].

CD39 is also upregulated in conventional CD4^+^ T cells in the TME, which have been shown correlated with impaired anti-tumor activity [43, 64, 75]. Consistently, silencing CD39 and/or CD73 increased anti-tumor activity of human CD4^+^ T cells against ovarian cancer cells. Furthermore, CD39 contributes to apoptosis of CD4^+^ T cells in vitro. CD39 expression is also increased in T cells during ageing [10].

### CD8^+^ T cells

CD39 is a checkpoint and exhaustion marker in CD8^+^ T cells [76–79]. Evidences showed that tumor-reactive CD39^+^CD8^+^ T cells isolated from human malignancies exhibit exhaustion phenotypes, including decreased production of IFN-γ, IL-2 and TNF-α and overexpression of several checkpoint receptors such as PD1, TIM3, LAG3, TIGIT and 2B4 [10]. Similar findings have been reported in lung cancer, colorectal cancer, head and neck cancer, breast cancer and liver cancer [36, 38, 76, 79]. In addition, CD39^+^ CD8^+^ T cells could also be detected in metastases of lymph nodes and other tissues [80]. Triggering T cell receptor alone induces CD39 expression on CD8^+^ T cells in peripheral blood mononuclear cells (PBMCs) breast cancer patients [10, 31]. Further evidence also revealed that clinical responses to ICB therapy was accompanied by accumulation of the CD39^+^CD8^+^ T cell population in the blood [81]. Besides tumor, chronic viral stimulation has also been linked to CD39 upregulation in CD8^+^ T cells [10, 82].

However, some studies suggest that CD8^+^ T cells expressing CD39 have regulatory functions [31, 83]. For example, CD39 has been found to be involved in regulating the inhibitory ability of tumor invasive CD8^+^ Tregs, and the isolated CD39^+^CD8^+^ T cells can indeed limit T cell proliferation in vitro [77]. In addition, CD39 antagonist significantly reduced the inhibitory ability of CD8^+^ Tregs, highlighting its key role in mediating inhibitory function, which also applies to CD4^+^ Tregs [65, 84]. These data suggest that antigen-induced CD39 upregulation activates the internal inhibitory function of CD8^+^ Treg cells, which helps to limits excessive immunopathological response. This regulatory mechanism could be beneficial in the situations where an overwhelming immune response is undesirable, such as following the recovery of infection. On the contrary, it can have a negative impact under tumor conditions by providing tumor-specific CD39^+^CD8^+^ T cells with inhibitory abilities [37, 38, 76, 80].

At present, little is known about the mechanism of the upregulation of CD39 in CD8^+^ T cells under specific circumstances [31]. Compared with circulating cells, the expression of CD39 in tumor-infiltrating CD8^+^ T cells increased significantly, suggesting that special factors in TME may be responsible for the rise in CD39 level [37, 80]. Cytokines such as IL-6 and TGF-β may contribute to the CD39 upregulation in tumor-infiltrating CD8^+^ T cells [10]. Expression of CD39 on long-lived memory CD8^+^ T cell subsets may have distinct cellular metabolic profiles and promotes survival [77]. Furthermore, eATP/CD39 can modulate mammalian target of rapamycin (mTOR) activation which regulates stress resistance and organism longevity [46, 85]. Hence, it is feasible that regulation of the CD39 pathway and purinergic signaling will affect T cell survival [53].

Based on the expression and function of CD39 on T cell memory and exhaustion, these findings imply that targeting CD39 might represent a novel strategy for the treatment of cancer [10].

## CD39 and NK cells

CD39 expression is at low level in NK cells [20, 21, 68, 86]. However, CD39 and CD73 are upregulated in lung tumor-infiltrating NK cells [9, 86]. CD39-mediated immune-suppression of NK cells has recently been reported in various tumor metastasis models. First evidence was shown by utilizing the CD39 inhibitor sodium polyoxotungstate (POM1). The anti-metastatic activity of POM1 was completely dependent on the presence of NK cells and CD39 expressed by hematopoietic cells. There is also observed remarkable decrease in experimental lung metastases in CD39-deficient mice compared with WT mice, with mechanisms of NK cells and IFN-γ-dependent manner. Although adenosine can directly suppress NK cells, it cannot rule out an indirect mechanism involving NK/myeloid cell crosstalk which could also affect NK cell function in tumor settings [86].

A monoclonal antibody specific to CD39 that blocks ecto-enzyme CD39 can suppress both experimental and spontaneous metastases in mice. Furthermore, the CD39-targeting monoclonal antibody show higher antimetastatic activity than the CD39 inhibitor POM1 and antagonists that block other members of the adenosinergic family (e.g., CD73 and A2AR). The CD39-targeting monoclonal antibody relied on myeloid cell -expressed CD39 and NK cell effector function to control metastases, while NK cell CD39 expression was not required [9].

## Targeting CD39 in cancer (Mechanisms of action)

Targeting adenosine pathway has shown tremendous promise for tumor immunotherapy [7, 11, 87–89]. In the last decade, numerous studies have revealed that antagonists that block the direct adenosine-generating enzyme CD73 and the adenosine receptors A2AR or A2BR in cancer have a strong anti-tumor effect by restricting the adenosine-induced immune-suppressive microenvironment [90–93]. More than 30 clinical trials targeting CD73 or A2AR/A2BR in cancer are now underway [10, 12, 31]. More recently, study utilizing the CD39 pharmacological inhibitor POM-1 has described therapeutic potential of targeting CD39 in cancer [86]. However, doubts concerning the specificity, pharmacokinetics, therapeutic half-life, and toxicity of the CD39 pharmacological inhibitors prevent its further clinical applications. Thus, effective anti-mouse and anti-human CD39 mAb reagents have been developed in the recent four years and showed therapeutic potential in solid tumors, either as monotherapy or in potential ICB combinations [9, 27, 47, 94]. Moreover, targeting CD39, the rate-limiting ecto-enzyme of the ATP-adenosine pathway, demonstrated more profound anti-tumor effects compared with antagonists that block other members of the adenosinergic family (e.g., CD73 and A2AR/A2BR) [9, 14, 26]. Although the potential for targeting CD39 in cancer is promising, the mechanisms of action are still partially mysterious and need further investigation.

Targeting CD39 offers the theoretical potential to impact anti-tumor immunity in a twofold fashion: firstly, decreasing the extracellular adenosine reverses the adenosine receptor-mediated broadly and long term immune-suppressive TME; secondly, preventing the conversion of ATP to AMP results in the accumulation of eATP, a pro-inflammatory danger signal which will be available at relatively high concentration and thus stimulates immune cells in the TME [95–99].

**Fig. 3:**
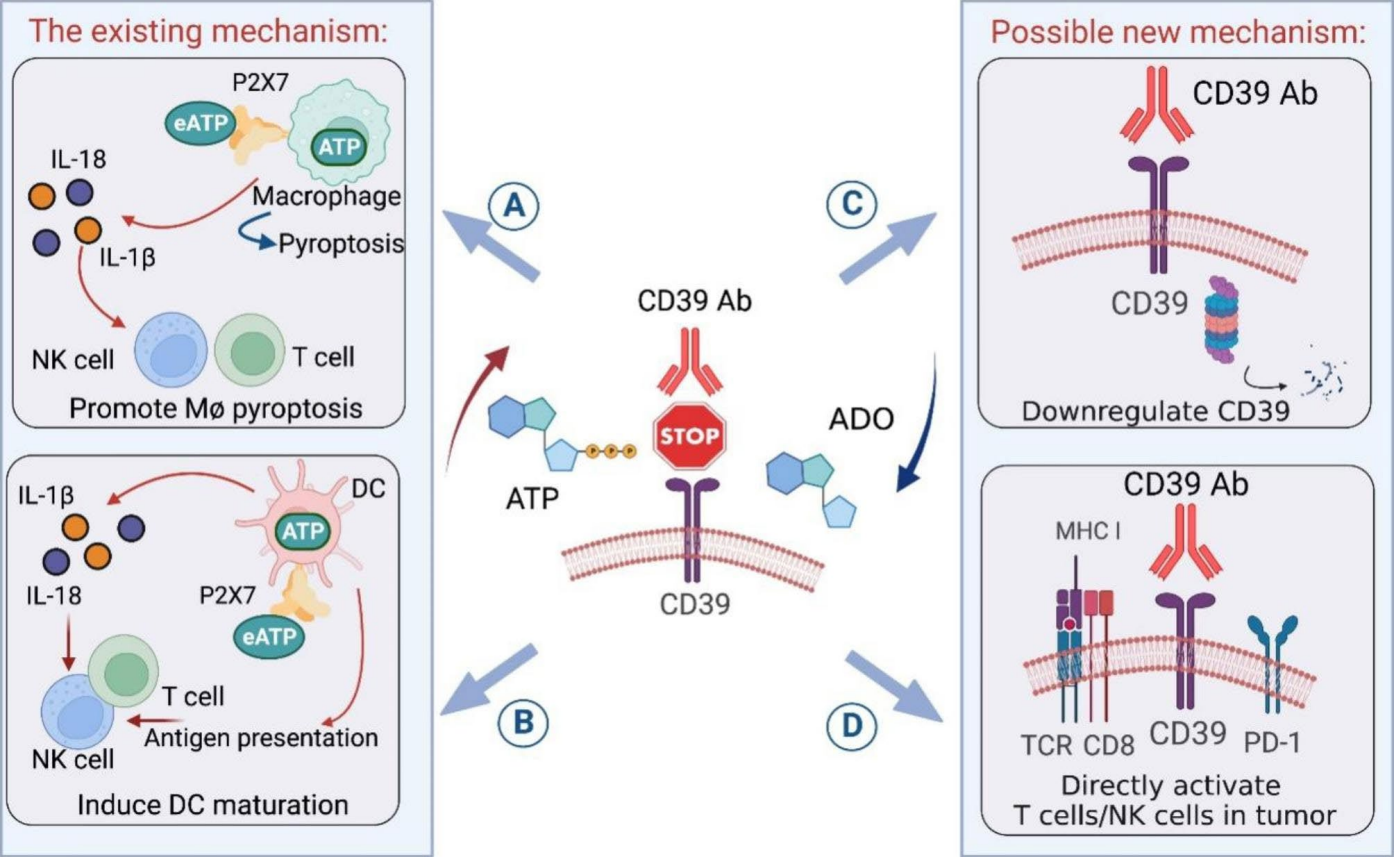
The mechanisms of action of targeting CD39 in cancer. CD39 blocking antibody can increase the level of extracellular ATP (eATP) in the tumor microenvironment (TME) by inhibiting ATP degradation by CD39. The increased eATP levels activate the P2 × 7 receptor on macrophages and dendritic cells (DCs), resulting in inflammasome-mediated release of pro-inflammatory cytokines, including IL-18 and IL-1β, which support effector T cell and natural killer (NK) cell-mediated cytotoxicity. The increased eATP levels not only induce pyroptosis in P2 × 7 + macrophages (A) but also bind to P2 × 7 on DCs, enhancing antigen presentation and maturation, further supporting effector T cell and NK cell-mediated cytotoxicity (B). These are the two known mechanisms of action of targeting CD39 in cancer. Additionally, targeting CD39 may downregulate CD39 expression by binding to the CD39 receptor (C) and directly activate CD39 + tumor-specific T cells or CD39 + NK cells (D), which could be two potential new mechanisms

Although targeting CD39 with a monoclonal antibody that blocks the mouse ecto-enzyme activity reverses the ATP/adenosine pathway in the TME, there are still limited understanding of how the immune cells respond to CD39 targeting therapy. Here we would like to summarize the possible immune cell reactions as potential mechanisms of action for targeting CD39 in cancer [26, 28, 87, 100] (Fig. 3). As further studies are emerging, rational design of targeting CD39 in cancer is expected.

1. **Activation of the inflammasome in macrophages.** Adenosine directly interacts with macrophages by binding to adenosine receptors, which impairs the differentiation and maturation of macrophages as well as lowers the pro-inflammatory mediators and enhances pro-tumorigenic markers [5, 55]. As a result of the reduced adenosine accumulation caused by blocking CD39, the anti-tumor activity of macrophages will be promoted. Furthermore, ATP accumulation in the TME by CD39 inhibition promotes pyroptosis and inflammatory response of macrophages via a P2 × 7-dependent manner. Moreover, the anti-tumor efficacy of CD39 inhibition is also amplified by intratumoural IL-18 expression by macrophages [10, 26].
2. **Increasing APC maturation and antigen presentation.** The antigen-presenting capacities and DC maturation are crucial for sustained anti-tumor immunity. Adenosine directly inhibits DC maturation and diminishes DC capacity to prime Th1 cell immunity, resulting in the decreased production of the pro-inflammatory cytokines IL-12 and TNF through the A2AR and/or A2BR pathways on DCs. Furthermore, it was reported that human monocyte-derived DCs exhibit an enhancement of maturation via increasing dosages of eATP using an allosteric modulator of CD39. Similarly, treatment of myeloid-derived DCs with ATP and a CD39 ecto-enzyme activity inhibitor resulted in an upregulation of CD86 expression and inflammatory cytokine production [28, 87]. In addition, POM-1 treatment induced the expansion of cDC1 in the TME in a mouse bladder cancer model, while *Batf3*^*-/-*^ cDC1 -deficient mice failed to respond to POM-1 treatment [101]. These indicated that targeting CD39 might promote cDC1 -dependent tumor antigen presentation for anti-tumor CD8^+^ T cell activation.
3. **Downregulating cell surface CD39.** CD39 expression is increased in almost all cell types, including tumor cells, endothelial cells, fibroblasts, and immune cells in the TME. CD39 ecto-enzyme inhibitors have already demonstrated perfect functional activity in blocking CD39 phosphohydrolytic processing of ATP. Interestingly, human CD39 mAb significantly downregulates CD39 on both tumor cells and tumor-infiltrating lymphocytes by immunohistochemistry and flow cytometry studies, suggesting a new mechanism of action involving additional downregulation of CD39 on cell surface with CD39 targeting antibodies [10, 26].
4. **Directly activating exhausted CD8**^**+**^**T cell.** Targeting CD39 can improve the anti-tumor efficacy with various immune cells and non-immune cells involved. Hence, determining whether CD39 inhibition, especially with antibodies, can directly activate exhausted CD8^+^ T cells in the TME is difficult. CD39 is an exhaustion marker that co-expresses with PD-1 in tumor associated CD8^+^ T cells. CD39 inhibition with a small-molecule or an antibody can increase the proliferation and function of both CD4^+^ and CD8^+^ T cells in vitro. Moreover, CD39 inhibition with a small molecule or an antibody can strongly activate exhausted CD8^+^ T cell in the TME. However, more in vivo evidence of the direct activation of CD8^+^ T cells by CD39 targeting in tumor is still needed [10, 12, 26, 31].
5. **Activating exhausted NK cell.** In addition to CD8^+^ T cells, targeting CD39 by POM-1 or an antibody also increased the proportion of NK cells and more mature CD11b^+^ NK cells in the TME, as well as promoted expression of IFN-γ and CD107a by tumor-infiltrating NK cells [9, 86, 101], suggesting that targeting CD39 might also benefit the functional recovery of tumor -infiltrating NK cells.

## CD39 and PD-1 combination therapy

CD39 and PD-1 are highly co-expressed in both CD4^+^ and CD8^+^ T cells in the TME [10, 12, 26]. Both CD39 and PD-1 are perfect immunotherapy targets for tumor control since they can define the exhaustion phenotype of T cells [31, 38, 76]. Unlike PD-1, CD39 is also upregulated in almost all cells in the TME, including tumor cells, endothelial cells, fibroblasts, and immune cells [10, 12]. The mechanisms of action of ICB for PD-1 mostly rely on the conversion of exhausted T cells to activated T cells in tumor. However, as previously indicated, the mechanisms of action of antagonists that target CD39 are quite complex and almost completely different from ICB for PD-1. Previous research has demonstrated that combined treatment with anti-CD39 and anti-PD1 is more effective in combating tumors than either treatment alone [10, 26]. Anti-CD39 can convert anti-PD1 resistant tumors to sensitive, thereby transforming “cold” tumors to “hot” tumors. Mechanistically, anti-CD39 increases the proliferation of tumor infiltrating lymphocytes, while anti-PD-1 reverses the exhaustion phenotype of these lymphocytes. Together, these treatments achieve a synergistic antitumor effect. Hence, a synergistic anti-tumor effect of combination therapy with ICB for PD-1 and antagonists for CD39 is expected in clinical trials.

## Perspective

CD39 is a novel biomarker of exhausted T cells and an immune checkpoint target for tumor immunotherapy [76, 79, 82, 94]. The immune-suppressive effect of CD39 is mainly mediated by promoting the production of adenosine. CD39-targeted monoclonal antibodies have been developed in the last four years and have shown to significantly suppress tumor growth in preclinical cancer models [27, 28, 94]. However, most investigations on CD39 targeting in cancer are still limited to mouse tumor models. Moreover, more research into the mechanisms of action of targeting CD39 is required.

More than 50 therapeutic combination therapies targeting the ATP–adenosine pathway via CD73 or A2AR/A2BR antagonists are being explored in the clinic [3, 10, 14]. The combination therapy trials in clinical landscape are under rapid evolution. Hence, prioritizing strategies that offer a combinatorial benefit when targeting CD39 in cancer is critical. Recently, several CD39-targeting agents have entered clinical trials (Table 1), with additional agents set to follow [11, 28, 87, 94]. As first-in-human studies are now underway, further insights into the mechanisms of action and the sensitive tumor types with anti-CD39 therapy are anticipated in the coming years.

**Table 1:**
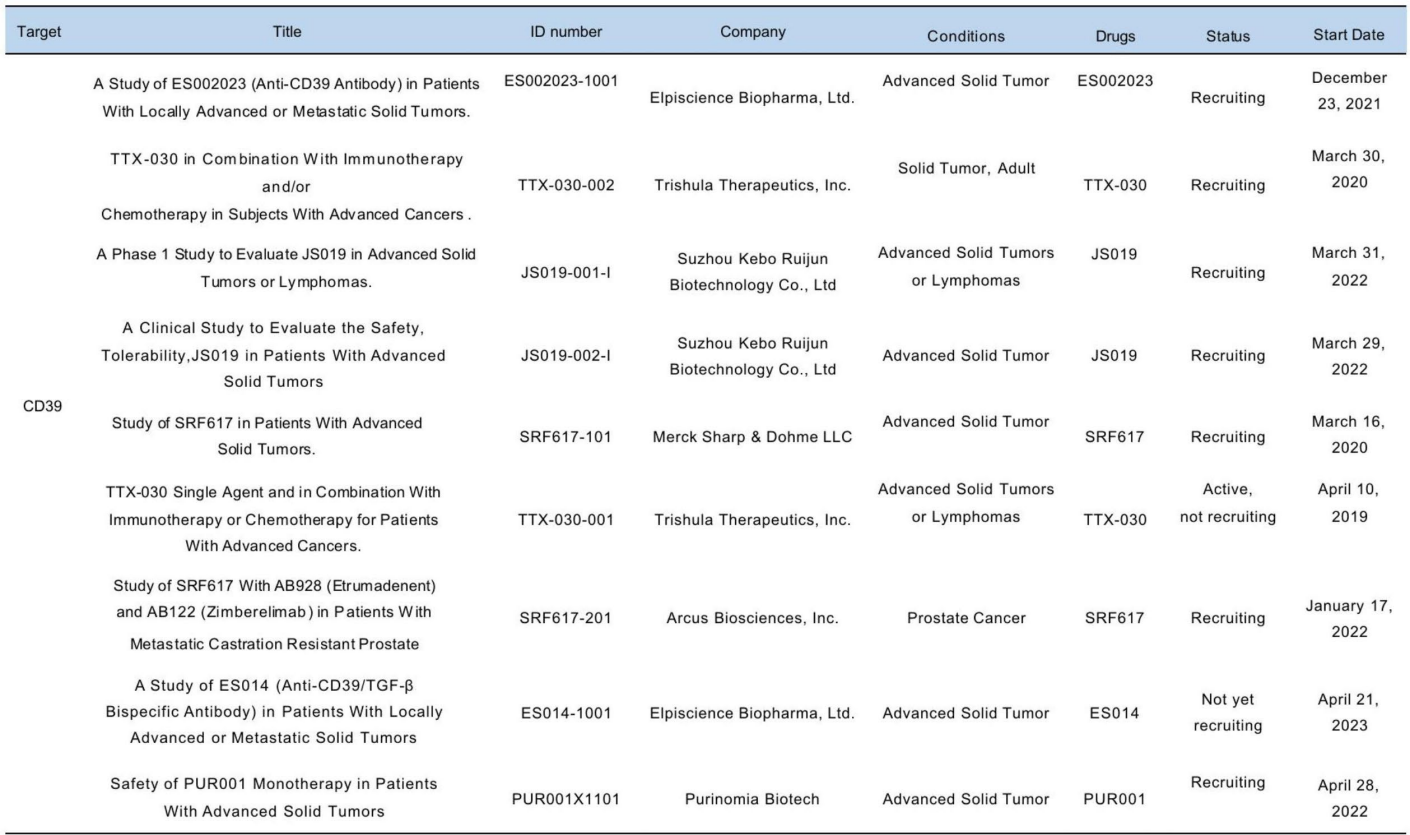
Targeting CD39 in clinical trials

## Data Availability

Not applicable.

## Abbreviations

ATP: Adenosine triphosphate
ADP: Adenosine diphosphate
AMP: Adenosine monophosphate
cAMP: Cyclic Adenosine monophosphate
TME: tumor microenvironment
eATP: extracellular ATP
CD39: Ectonucleoside triphosphate diphosphohydrolase-1
CD73: Ecto-5′-Nucleotidase
A2AR: Adenosine A2a receptor
A2BR: Adenosine A2b receptor
PD-1: ProgrammedDeath-1
ICB: Immune checkpoint blockade
GPCR: G protein coupled receptors
NAD+: Nicotinamide adenine dinucleotide
NT5E: Ecto-5-nucleotidase
ENPP: Ecto-nucleotide pyrophosphate/phosphodiesterases
ENTPDases: Ectonucleoside triphosphate diphosphohydrolases
TNF-α: Tumor necrosis factor-alpha
AHR: Aryl hydrocarbon receptor
LPS: Lipopolysaccharide
IL: Interleukin
Tregs: T regulatory cells
MDSC: Myeloid-derived suppressor cells
NSCLC: non-small-cell lung cancer
FOXP3+: Forkhead box protein 3
IFN-γ: Interferon-γ
MS: Multiple sclerosis
TR1: Type 1 regulatory T
PBMCs: Peripheral blood mononuclear cells
mTOR: Mammalian target of rapamycin
